# Modelling the effect of cell motility on mixing and invasion in epithelial monolayers

**DOI:** 10.1007/s10867-024-09660-8

**Published:** 2024-07-20

**Authors:** Faris Saad Alsubaie, Zoltan Neufeld

**Affiliations:** School of Mathematics and Physics, The University of Queensland, St Lucia, Brisbane, 4072 Queensland Australia

**Keywords:** Cell motility, Cell competition, Invasion wave, Cellular Potts model

## Abstract

Collective cell invasion underlies several biological processes such as wound healing, embryonic development, and cancerous invasion. Here, we investigate the impact of cell motility on invasion in epithelial monolayers and its coupling to cellular mechanical properties, such as cell-cell adhesion and cortex contractility. We develop a two-dimensional computational model for cells with active motility based on the cellular Potts model, which predicts that the cellular invasion speed is mainly determined by active cell motility and is independent of the biological and mechanical properties of the cells. We also find that, in general, motile cells out-compete and invade non-motile cells, however, this can be reversed by differential cell proliferation. Stable coexistence of motile and static cell types is also possible for certain parameter regimes.

**Supplementary Information:**

The online version contains supplementary material available at 10.1007/s10867-024-09660-8.

## Introduction

Cell motility plays an essential role in a range of biological processes such as embryonic development, wound healing, cancer metastasis, and tissue vascularisation [1–4]. At a single cell level, the mechanisms of active cell motility have been studied extensively and a range of mathematical and computational models exist that describe how actomyosin networks in cell protrusions generate active cell movement [5–7]. The direction of cell movement is regulated by external and internal biochemical signals that generate the asymmetric, i.e. polarised, structure of the moving cell [8]. The process of cellular chemotaxis involves sensing of external concentration gradients resulting in cell polarization. This can be described through feedback mechanisms in a nonlinear reaction-diffusion system of intracellular signalling proteins [9–11].

In multicellular organisms a large number of interacting motile cells may produce emergent behavior [12–14]. A well-known example of such collective cell movement is the migration of neural crest cells during embryo development [4, 15–17]. Collective motility in epithelial layers, where the cells are directly connected to each other, was also studied using computational models [18, 19]. Collective movement and wave propagation in such cell layers were found to be dependent on the mechanical properties of the cells [20, 21] regulated by the contractility of the actomyosin cortex.

In this paper, we investigate the role of cell motility in an epithelial cell layer composed of two cell types with different properties that compete for space. Previous work has demonstrated that competition of non-motile cells, with different division rates or different mechanical properties, can lead to invasion of winner cells as a propagating front while the other cell type is eliminated [22]. Here, we extend this computational model to study how active cell motility influences the outcome of competitive cell invasion. This kind of competition between motile and non-motile cells may arise when mutant cancer cells invade normal tissue.

In the following section, we present the computational model for epithelial cell competition combined with the dynamics of cell polarization, which is adapted from previous work on collective migration [21]. Then, the numerical results are presented in two separate sections. First, we consider the mixing of cells at the interface between two cell types when the cell numbers of each type are fixed. Then, we investigate the more general case, relevant for longer-term behavior, where cell division and death are included which may lead to competitive invasion.

## Model description

To model the effect of cell motility on epithelial invasion, we propose a 2-D computational model that represents both motile and non-motile cells within a monolayer. The model is based on the cellular Potts model (CPM) such that every cell covers a set of connected lattice sites (or pixels), where each pixel can only be occupied by one cell at a time. The movement of the cell boundaries is determined by minimising the energy function [21–23]:

1 $$\begin{aligned} E = \lambda _{\text {area}} \sum _{\alpha =1}^{N}\left( A_{\alpha }-A_{0}\right) ^{2}+ \lambda _{\text {cont}}\sum _{\alpha =1}^{N} L_{\alpha }^{2}+ & \sum _{\overrightarrow{i},\;\overrightarrow{j}} \phi \left(\alpha _{\;\overrightarrow{i}}\;,\alpha _{\;\overrightarrow{j}}\right) \left( 1-\delta (\alpha _{\;\overrightarrow{i}}\;,\alpha _{\;\overrightarrow{j}}\,)\right) + \nonumber \\+ & \sum _{\alpha =1}^{N_{m}} \overrightarrow{F_{\alpha }}\cdot \overrightarrow{r_{\alpha }} \end{aligned}$$

where $A_{\alpha }$ is the cell area and $L_{\alpha }$ is the perimeter of each cell $\alpha$ of N cells ($\alpha =1,2,...,N$). The first three terms of the energy function (1) describe mechanical properties and interactions of the cells, whereas the last term represents a motile force driving the cells into the direction of $\overrightarrow{F_{\alpha }}$, and $\overrightarrow{r_{\alpha }}$ is the position vector of the cell’s center of mass [19]. We denote the total number of motile cells as $N_{m}$.

The elementary step of the CPM is defined as a small perturbation among the pixels on the boundaries of the cells when the cell index is changed to a neighbouring cell. Then, the energy change resulting from this perturbation is computed. If the perturbation leads to a decrease in effective energy, it will be accepted unconditionally. Otherwise, it will be accepted with a probability that decreases exponentially with the change of energy [22, 23]. The unit time of the CPM model is the Monte Carlo step (MCS) corresponding to an attempt to change the cell index of every lattice site by the elementary step described above.

Motile cells establish an asymmetric spatial distribution of intracellular signaling molecules that regulates the dynamics of cell protrusions and generates motility [21, 24–26]. We characterise the magnitude and direction of this asymmetry by the polarisation vector $\overrightarrow{\rho _{\alpha }}$. Following [21, 27] the polarisation dynamics of motile cells is described by an ordinary differential equation coupled to each cell:

2 $$\frac{d\overrightarrow{\rho _{\alpha }}}{dt}= {\zeta\overrightarrow{v_{\alpha}}}- {\gamma\overrightarrow{\rho _{\alpha}}}$$

where $\overrightarrow{v_{\alpha }}$ is the velocity vector of the cell’s center of mass and $\zeta$ is a scaling factor. When a polarised cell is unable to move, e.g. due to an obstacle, its polarisation is gradually lost. The parameter $\gamma$ is the inverse persistence time of cell polarisation. The first term of (2) represents the effect of reinforcement of polarisation due to actual movement, whereas the second term describes the decay of $\rho _{\alpha }$ when a cell stops moving [18]. It has been observed experimentally that epithelial cells can switch between stationary and migrating states [28, 29]. Therefore, we assume that the magnitude of the motile force is described by a Hill function [21]:

3 $$\begin{aligned} \overrightarrow{F_{\alpha }} = F_{M} \frac{\overrightarrow{\rho _{\alpha }}}{|\overrightarrow{\rho _{\alpha }}|}\frac{|\overrightarrow{\rho _{\alpha }}|^{\mu }}{|\overrightarrow{\rho _{\alpha }}|^{\mu } + \beta ^{\mu }} \end{aligned}$$

where $F_{M}$ is the maximal motile force and $\beta > 0$ is a half-saturation constant, so that $|\overrightarrow{\rho _{\alpha }}| = \beta$ corresponds to a partially polarised cell that generates half of the maximum motile force.

**Fig. 1:**
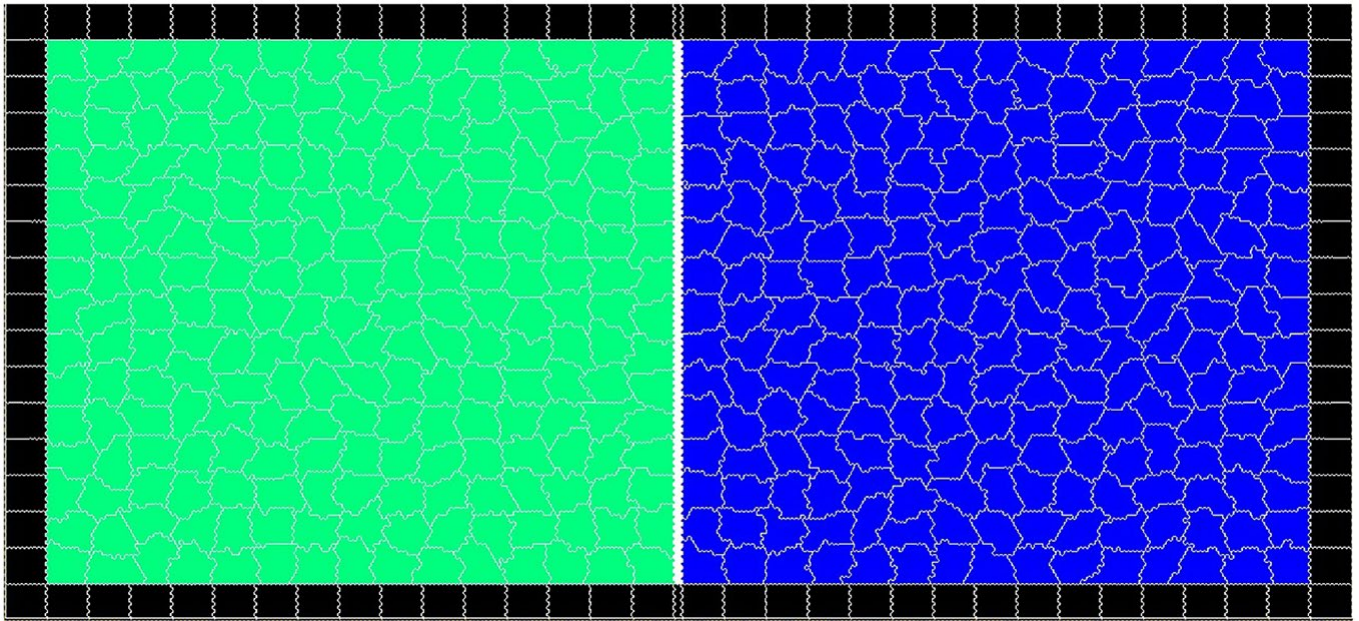
Snapshot of the initial stage of the simulations illustrating the representation of the epithelial monolayer on a rectangular domain before the barrier is removed ($t=400\;MCS$). The vertical white line indicates the barrier between motile (green) and non-motile cells (blue). The black cells are fixed rectangles and form a wall which maintains the cells in the domain during the simulations. The cell-cell adhesion parameter is $\phi = -10$ for all cells and is set to zero between the wall and cells. The cell parameters are: $\lambda _{area}=70, \lambda _{adh}=-10$, $\lambda _{cont}=0.5$

We will use this model to investigate the outcome of bio-mechanical interactions between two spatially separated epithelial cell populations, where one cell type is motile ($F_m > 0$) while other cells are not able to generate motility ($F_m = 0$). We consider a two-dimensional domain of $302 \times 150$ pixels with a solid impermeable boundary. Initially, each half of the domain is covered by a confluent layer of cells with an equal number of motile and non-motile cells, $N_{m}=N_{nm}=225$ separated by a 2-pixel wide barrier (Fig. 1). The initial area of the cells was set to the target area $A_{0}=100$ pixels. In the first stage of the simulations, the cell motility is switched off until the cells reach their equilibrium shapes, typically around 400 MCS. It has been shown previously that, depending on the relative strength of cell cortex contractility and cell-cell adhesion, the cells may produce relatively static quasi-hexagonal shapes, known as the “hard” regime, where the cell perimeter is close to minimal [27, 30]. In the opposite case, cell-cell adhesion dominates over the cortex contractility resulting in a “soft” regime, where the cells have dynamically moving protrusions and longer perimeters.

After the initialisation of cell shapes the motile cells in the left side of the domain are allowed to spontaneously polarise according to the Eq. (3), that typically leads to a streaming motion when the contractility of the cell perimeters is not too strong [21].

We will consider two types of simulations. In the first case, we investigate the mixing between motile and non-motile cells after the barrier is removed and monitor the number of cells of each type crossing the barrier. In these simulations, the total number of cells in each sub-population, i.e. motile or non-motile, remains constant $N_{m}=N_{nm}=225$.

In the second case, we include cell division and death in the model and simulate the competition of two cell types for space over a longer time. The turnover regulates the homeostatic balance of the cell populations resulting in dynamically changing cell numbers.

To represent cell division and death in the CPM, we follow our earlier model of cell competition and invasion without motility [22]. Cell proliferation is linked to cell growth [31, 32], and therefore, if the cell area grows, the probability of cell division increases [33]. We assume that cell division is represented as a series of random events allowing individual cells to divide in each MCS with a fixed probability *B* if their area is larger than the target area, $A_{\alpha } \ge A_{0}$. The cells selected for division are separated into two new cells with an area equal to half of the original cell. Driven by the energy function of the CPM (1) the new daughter cells start to grow approaching the target area $A_{0}$. We also assume that motility stops during cell division; therefore, the polarisation vector is reset to zero. However, after division, random movements of cell boundaries typically lead to gradual spontaneous re-polarisation of the daughter cells.

Cell death is also modelled as a stochastic process and we assume that this is independent of cell size. Experimentally, it is observed that in dying cells the cortical cytoskeleton network becomes depolymerized [34, 35], and the connections of the dead cell with surrounding cells become incoherent [36], resulting in the degeneration of cellular components and the loss of cell mass [36, 37]. To implement cell death in the model, we assume that in every MCS cells have a fixed probability *M* to be selected for death. Then, the parameters of the dead cell are modified by setting the cell-cell adhesion, contractility, and target area to zero. To avoid the accumulation of dying cells in the domain, we increase the area compressibility of dead cells to $\lambda _{area}=500$. This ensures that the dead cell shrinks and is gradually excluded from the layer to mimic cell extrusion observed in experiments.

In the simulations with cell division and death, the initialisation stages are extended allowing the cells to reach their equilibrium density before the separating barrier is removed. Then, the motile and non-motile cells compete for space within the finite domain. Since cell numbers vary over time, typically one cell type, the winner cells, gradually invades the space occupied by the other cells and eventually eliminates the loser cells completely. We monitor the speed of cell invasion characterised by the rate of change of the cell numbers of each type.

For computer simulations, we use the CompuCell3D multicellular modelling environment [38].

## Results

First, we analyse the mixing of a fixed number of cells when the cell division and death is not included in the model and investigate the effects of the motile force with varying mechanical properties of the cells. We then model the competition of motile vs. non-motile cells when they can invade the area occupied by the other type and investigate how changing the different biological and mechanical parameters determine the outcome of the competition and the speed of the invasion process.

### Mixing of cells

To examine how motility affects the mixing of two cell types, we analyse the behaviour of an epithelial layer varying mechanical cell properties and the motile force. In the first stage of the simulations the cells reach their equilibrium shapes, after that, the motile cells spontaneously polarise while their movement is still restricted by the barrier. Then, the barrier is removed and we monitor the number of cells that cross the midline of the domain. We consider the layer of cells well mixed when approximately half of the motile (or non-motile) cells are on the opposite side of the domain relative to their initial position. The simulations are run until the proportion of each cell type on the two sides stabilises.

**Fig. 2:**
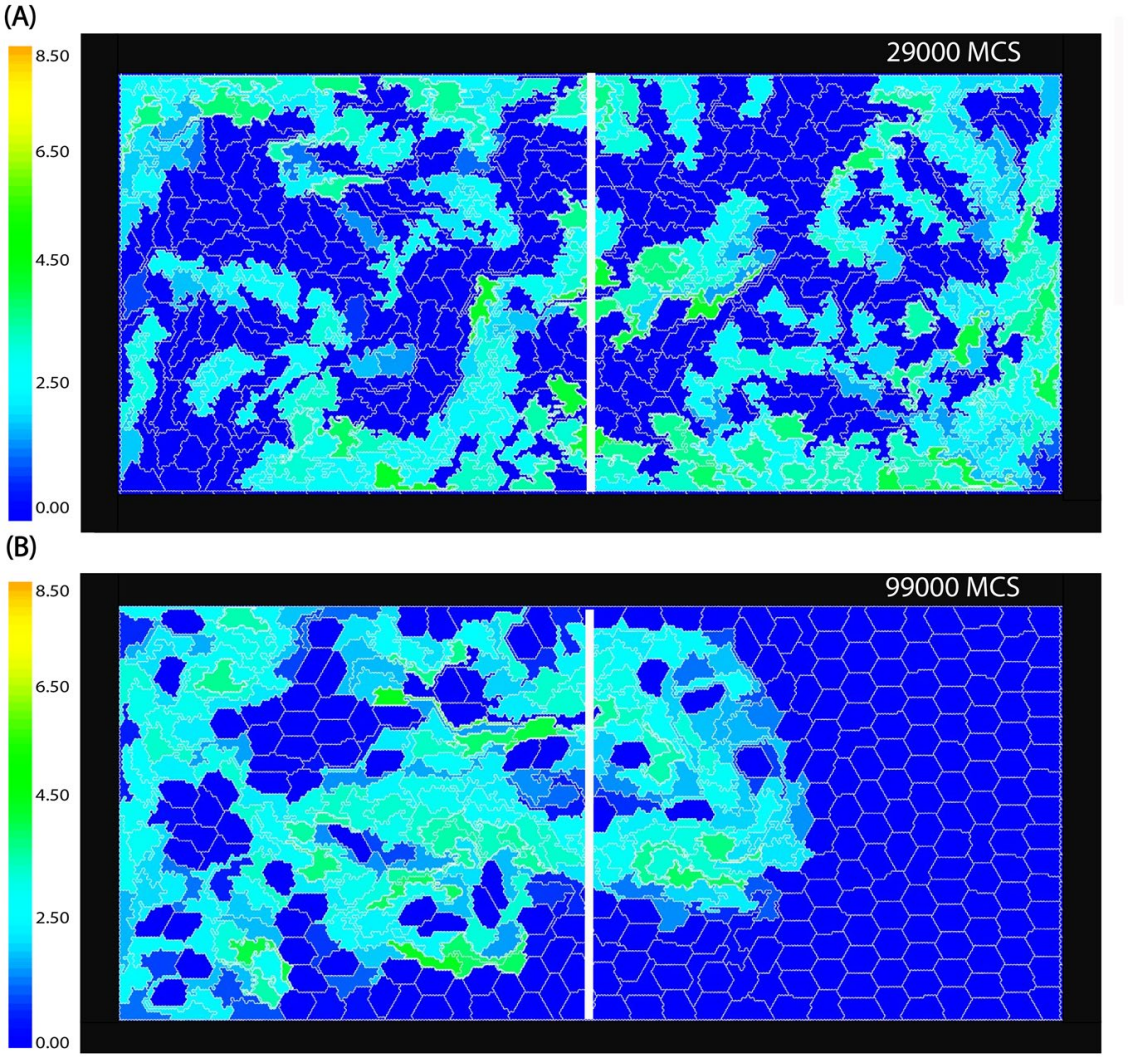
Snapshots of the spatial distribution of motile and non-motile cells, $t=25000 MCS$. **A** The motile cells (light blue or green) mix well with non-motile cells (blue) when the motile force strength is $F_{max}=1000$, and the contractility is low $\lambda _{cont}^m= \lambda _{cont}^{nm}=0.5$. See also Supplementary Movie 1 and Fig. 3 (red curve). **B** Partial mixing. The strength of the motile force was decreased to $F_{max}=400$ and the contractility was increased to $\lambda _{cont}^{nm}= 7$, and $\lambda _{cont}^{m}= 0.5$. About $32\%$ of motile cells crossed over to the right side by the end of the simulation. See also Supplementary Movie 2 and Fig. 3 (green curve). Color bar: the cell polarity magnitude ${\rho _{\alpha }}$

Two typical cell distributions are shown in Fig. 2 where the colour scale represents the magnitude of the polarisation vector $\rho _{\alpha }$ of each cell. Blue cells, with $\rho _{\alpha } =0$, are the non-motile cells that initially occupy the right half of the domain. Figure 2(A) shows a case of strong mixing where by the end of the simulation roughly half of the cells of each type are on both sides. This happens when the motile force $F_{max}$ is sufficiently large and the cell cortex contractility parameter $\lambda _{c}$ is relatively low. In Fig. 2(B), the motile force is lower, and the contractility of the cells is higher, resulting in more rigid cell shapes. In this case, the mixing is weaker and remains spatially restricted to the area around the initial interface; therefore, the proportion of cells that cross to the opposite side remains much lower (Table 1).

**Table 1:** The main model parameters used in the numerical simulations

Parameter	Value
The domain size	$302 \times 150$ pixels
Initial cell size	100 pixels
$\lambda _{\text {area}}^n$, $\lambda _{\text {area}}^{nm}$	70
$\lambda _{\text {cont}}^{nm}$, $\lambda _{\text {cont}}^{m}$ (soft/hard regime)	0.5, 7
$\lambda _{\text {adh}}^n$, $\lambda _{\text {adh}}^{nm}$	$-10$
$A_{\text {0}}$	100 pixels
Initial cell number per cell type	225
*T*	50
Half-saturation constant, $\beta$	1
Hill coefficient, $\mu$	10
Maximal motile force, $F_{max}$	Variable
Polarisation rate, $\zeta$	1
Depolarisation rate, $\gamma$	0.1

**Fig. 3:**
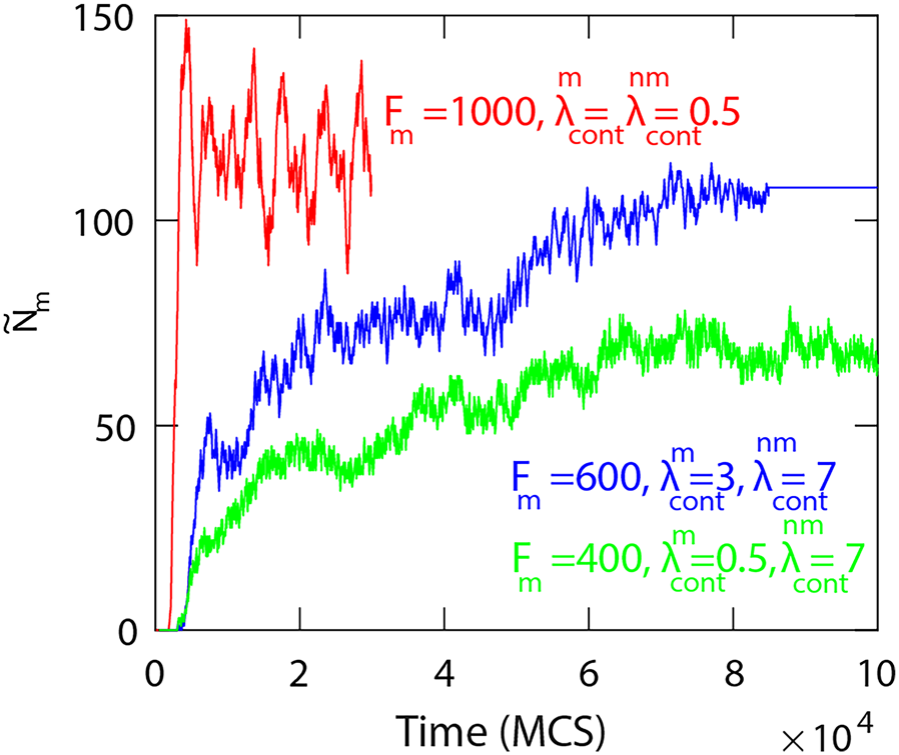
The number of motile cells that crossed into the right side of the mid-line versus time. High strength of the motile force and low contractility lead to enhanced mixing as shown by the red curve that saturates around $N_m/2 = 225/2$ cells where the contractility is $\lambda _{cont}^{nm}=\lambda _m=0.5$ and $F_m=1000$. Lower motile force and high contractility results in partial mixing when the number of cells crossing through the mid-line saturates at less than $50\%$ of cells, $\lambda _{cont}^{nm}=7$ (green curve) and $F_m = 400$. As the contractility of motile cells is increased to $\lambda _m = 3$ (blue curve), after 108 cells crossed to the opposite sides the motile cells lose their polarity and stop moving. The other parameters are fixed for all simulations at $\lambda _{\text {area}} = 70$, and $\lambda _{\text {adh}} = -10$

Figure 3 shows how the number of motile cells that moved onto the right side of the domain changes in time, for different values of the parameters. When the motile force is large and contractility is low, we see an example of fast and complete mixing across the domain (red curve). Intermediate motile force and stronger contractility results in partial mixing, where the proportion of cells that have crossed over to the other side saturates at around $30\%$ (green curve). When the contractility of motile cells is also increased the temporal fluctuations suddenly disappear at $t\approx 8 \times 10^4\;\text{MCS}$ (blue curve) indicating that the mixing of rigid motile cells into the non-moving cell population creates obstacles that can inhibit movement resulting in the complete loss of cell polarization of all cells.

These results show that cell motility is not the only factor that impacts on the mixing process between the cells, but the mechanical properties also play an essential role. When the cell boundaries are soft, due to low contractility or high cell-cell adhesion, the motile cells can more easily polarise and move. As a result, the mixing of cells is enhanced. However, if the contractility $\lambda _{cont}$ is high, the cells are in the so called hard regime so they are more rigid; therefore, mobility and mixing of cells are reduced [22, 27].

**Fig. 4:**
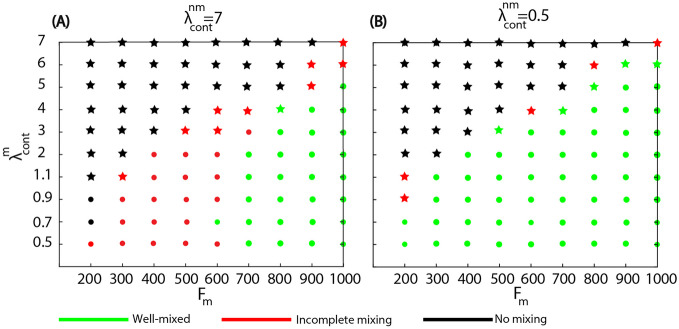
Phase diagram of the mixing between the two cell types while motile cell parameters are varied, and non-motile cells are in either the hard regime **A** due to strong contractility ($\lambda _{cont}^{nm} = 7$); or in the soft regime **B** when the contractility is low ($\lambda _{cont}^{nm} = 0.5$). The green dots or green stars indicate that the cell types mix well, i.e., at least half of the cells invade into the initial area of the other cell type. Black dots or black stars represent parameter combinations where there is no mixing and red symbols correspond to cases of partial mixing. The symbols indicate when the motile cells preserve (dots) or lose (stars) their polarization either during the simulation or fail to polarize

To obtain a detailed representation of the effect of parameters, we repeated the cell mixing simulations to consider cases when the mechanical properties of the two cell types are varied separately. Figure 4 shows the outcome of the mixing process as a phase diagram varying the maximum motile force along the horizontal axis and the contractility of the motile cells on the vertical. The simulations were performed for two fixed values of the contractility of the non-motile cells, corresponding to the hard and soft regimes, respectively.

We classify the simulations by following the number of motile cells that are located in the opposite half of the domain relative to their initial position, $\tilde{N}_m(t)$. While the number of cell types on each side fluctuates, we consider that the system is well mixed when at some point during the simulation at least half of the motile cells are fully in the right half domain, i.e. $\tilde{N}_m(t) \le N_m/2 = 225/2$. In the case of partial mixing $\tilde{N}_m(t) < N_m/2$ for all *t*, and the case of no mixing corresponds to $\tilde{N}_m(t) = 0$ when none of the cells cross the mid-line of the domain. The numerical results show better mixing of motile and non-motile cells in the soft regime than in the hard regime. We also observe that in the case of low motile force, or high contractility, the motile cells are either not able to polarise spontaneously, or they lose their polarization at a later stage after the barrier removal. These cases where polarization is lost in the final state are denoted by a star symbol in the phase diagrams.

### Competitive cell invasion

**Fig. 5:**
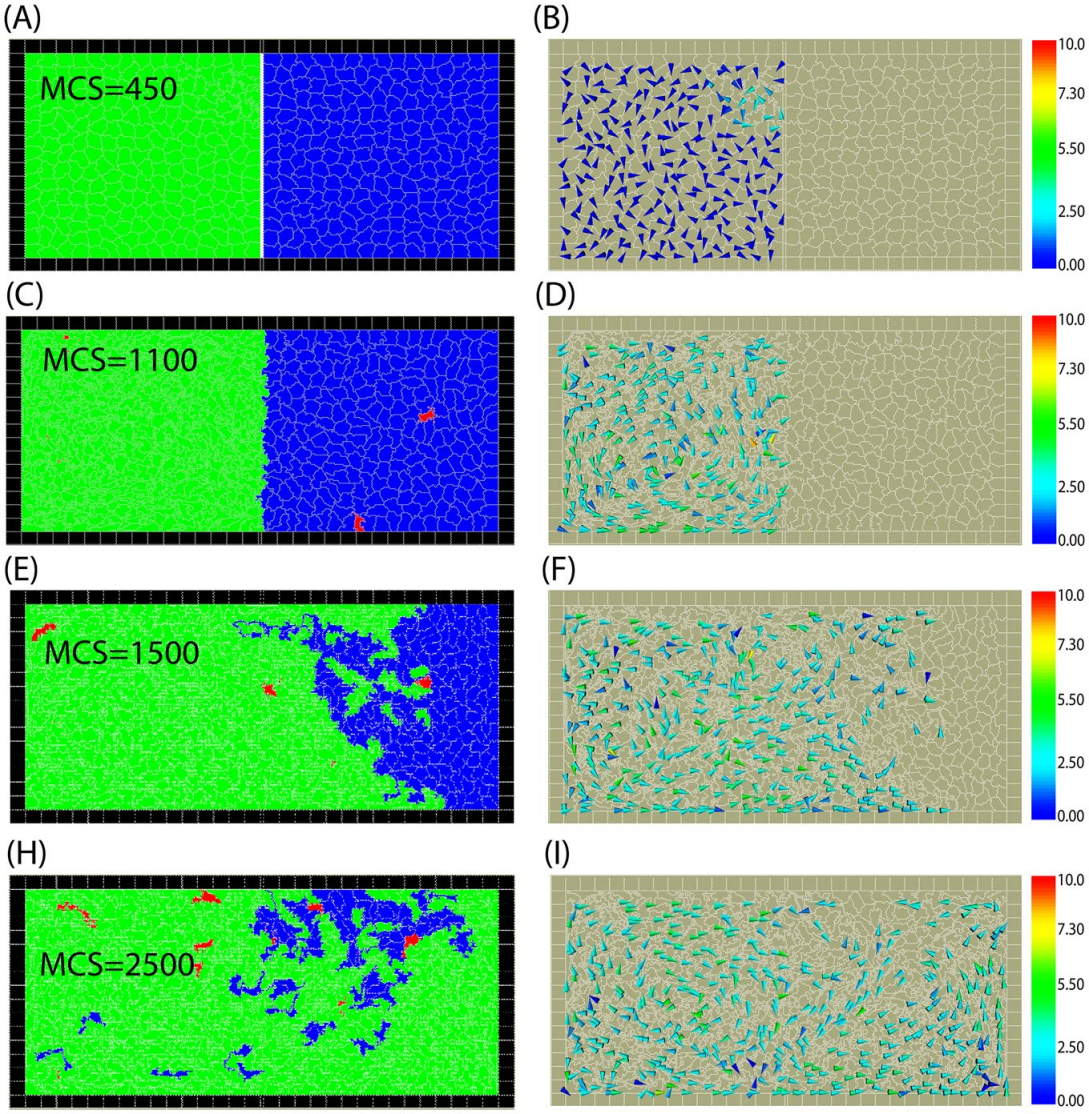
Snapshots from simulation of cell competition when motile cells invade the area of non-motile cells. In **A**, **C**, **E**, and **H** colours represent cell types (green: motile, blue: non-motile). In **B**, **D**, **F**, and **I** arrows show the direction of the polarisation of motile cells. **D** shows that cells produce streaming motion in their sub-domain even before the barrier is removed. **F** and **I** show the motile cells’ directions after the wall removal in early stage, and a later time of the invasion. Birth and death rates are $B_{m}=B_{nm}=0.1, M_{m}=M_{nm}=0.001$, and the mechanical parameters of all cells are $\lambda _{cont}=0.5,\lambda _{area}=70,$ and $\lambda _{adh}=-10$. Color bar: cell polarity magnitude $|{\rho }|$. See also Supplementary Movie 3

**Fig. 6:**
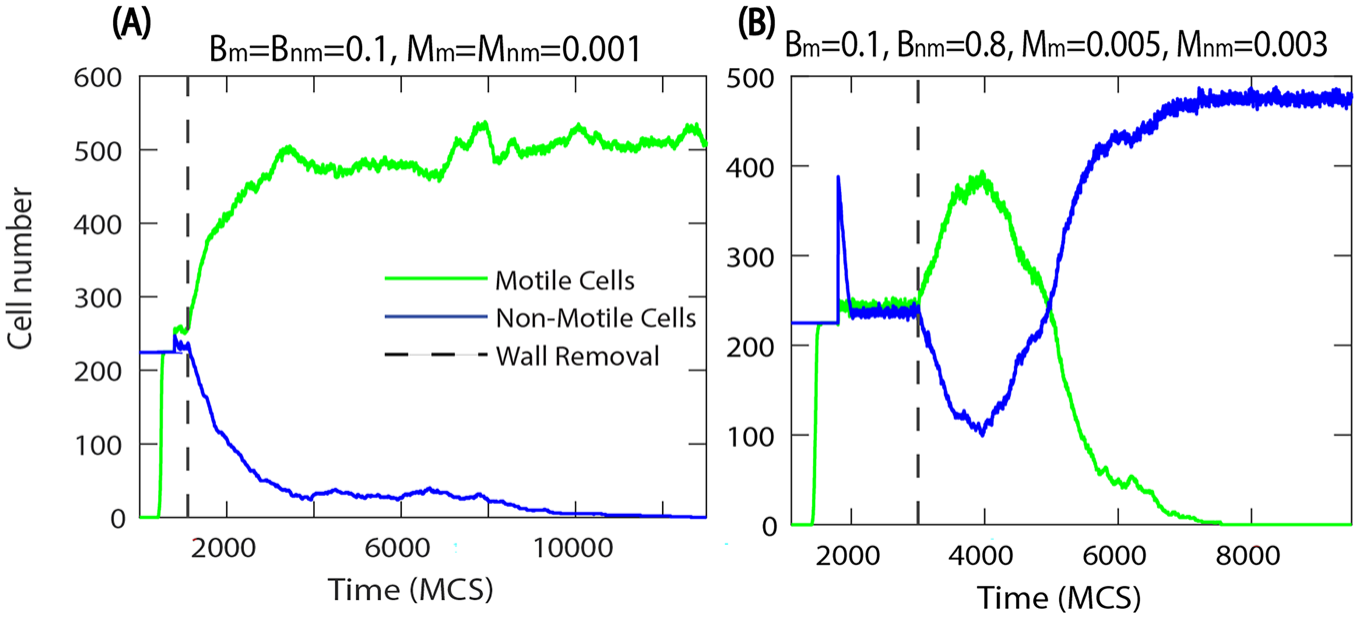
Number of motile and non-motile cells during the invasion. **A** motile cells invade, $F_{M}=1000$, the biological and mechanical parameters are the same for all cells, $\lambda _{cont}=0.5,\lambda _{area}=70,$ and $\lambda _{adh}=-10$. **B** temporary partial invasion of motile cells, which is then reversed, and finally non-motile cells win

Now, we consider the case when the cell numbers can change in time due to birth and death resulting in competition for space between the two cell populations. We modeled the competition of motile and non-motile cells initially occupying the left and the right halves of the domain. First, we considered the case when both cell types have the same mechanical and biological parameters and the only difference between them is motility. A typical example of the resulting invasion is shown in Fig. 5. The left panel shows the distribution of cell types (green: motile, blue: non-motile). The red regions indicate dead cells. We restrict the cell death rate to relatively small values so the proportion of dead cells remains low, i.e. below $\approx 5\%$ of all cells. The corresponding right panels in Fig. 5 show the direction and magnitude of the polarization vector of the motile cells. After the barrier is removed the area occupied by motile cells expands until non-motile cells are completely eliminated. This is also reflected in cell numbers plotted vs time in Fig. 6.

In comparison to the competition of different non-motile cells, described in [22], the shape of the interface appears to be more irregular and the front propagation rate is less uniform. Nevertheless, we can approximately quantify the propagation speed by fitting the average slope of the number of motile cells plotted as a function of time in the initial stage of the invasion and we convert this into propagation speed as

4 $$\begin{aligned} v = \frac{\Delta N}{ \Delta t} \frac{L}{ \rho _0} \end{aligned}$$

where $\rho _0$ is the equilibrium density of the invading cells, determined numerically as the average cell numbers per area when only a single cell type occupies the whole domain. *L* is the width of the domain, that we assume to be the length scale unit $L=1$.

**Fig. 7:**
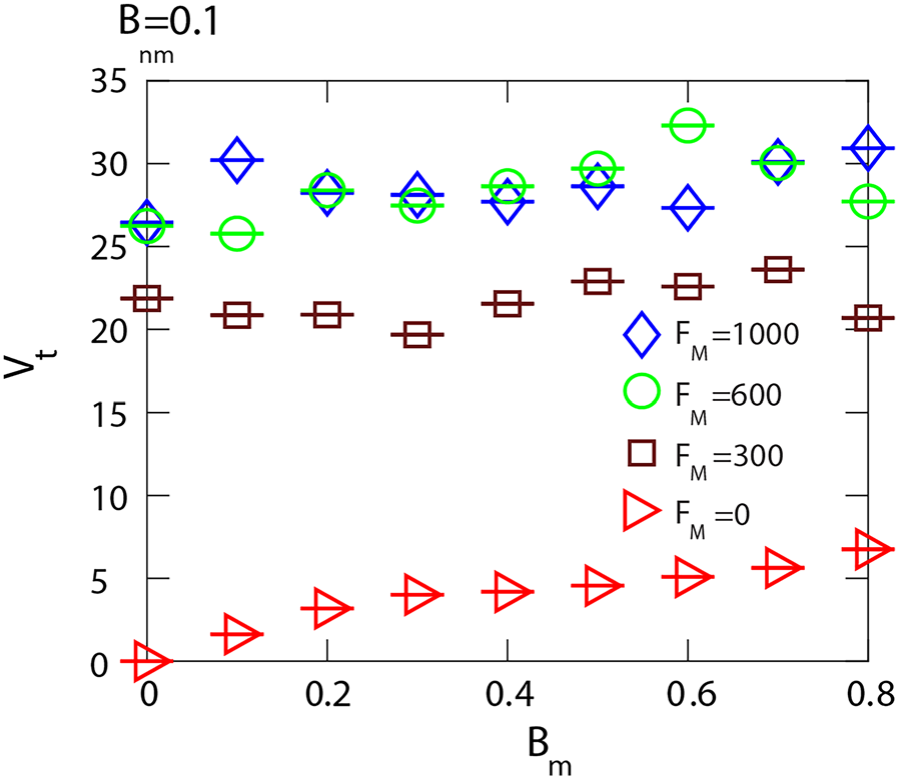
Invasion speed when the birth rates of motile cells are varied. In the case of high motile force $F_{max}=1000$, and 600, the invasion speed is not affected by the difference in birth rates (blue and green). However, for weaker motile force the invasion becomes slower, but still independent of the differential proliferation rates (brown squares). When motility is switched off $F_{max}=0$ the invasion speed increases monotonously with the difference of birth rates (red triangles). Birth and death rates are $B_{nm}=0.1, M_{m}=M_{nm}=0.001$, and the mechanical parameters of all cells are $\lambda _{cont}=0.5,\lambda _{area}=70,$, $\lambda _{adh}=-10$, and $F_{M}=1000$

From our simulations over a wide range of parameters, we find that when motile cells have equal or higher birth rates they can invade the whole system and eliminate non-motile cells. In order to investigate the combined effects of motility and biological differences on the invasion speed we varied the birth rate of motile cells. The results are shown in Fig. 7. The invasion velocity is primarily determined by motility and is not affected by biological differences between the cells. This is in contrast with the competition of non-motile cells ($F_{max}=0$) where we found previously that the invasion velocity increases monotonously with the difference between birth rates [22]. However, the invasion velocity decreases when the strength of the motile force, $F_{max}$, is reduced. For high motile force the invasion velocity saturates for $F_{max}=600$ indicating that the invasion rate is also limited by the cell turnover rate.

**Fig. 8:**
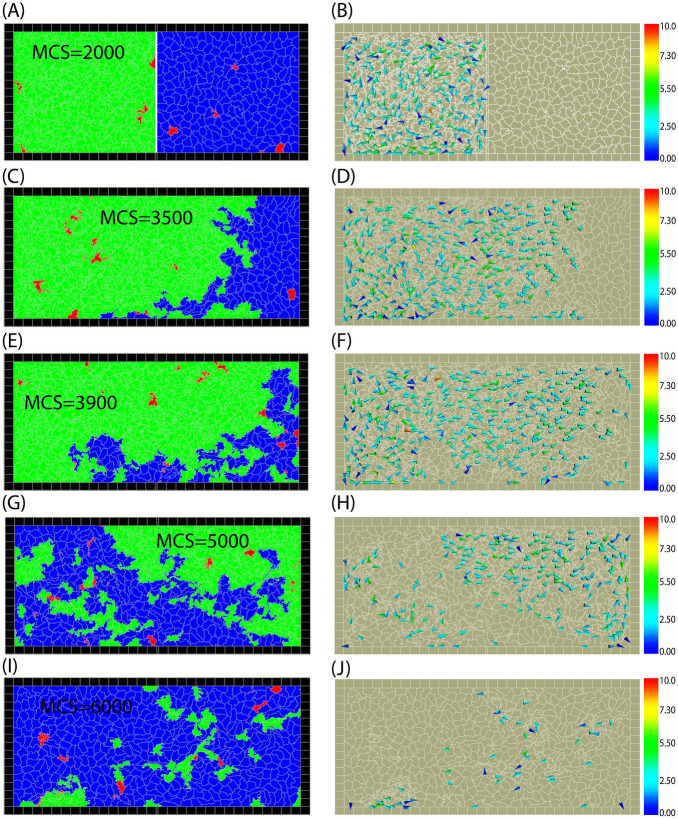
Snapshots from simulation where non-motile cells win. The cell parameters are $B_{nm}=0.8, B_m = 0.1, M_m=0.003, M_m=0.005$. First motile cells invade; however, non-motile cells later revert the invasion process and gradually occupy the whole domain while motile cells are eliminated. The mechanical parameters of all cells are the same: $\lambda _{cont}=0.5,\lambda _{area}=70,$ and $\lambda _{adh}=-10$, $F_{m}=1000$. Color bar: the cell polarity magnitude $|{\rho }|$. See also Supplementary Movie 4

Next, we investigated whether non-motile cells can invade motile cells if their disadvantage is compensated by higher birth rate and/or lower death rate. Snapshots from an example where non-motile cells are the winners are shown in Fig. 8. The corresponding cell numbers vs. time in Fig. 6(B) show that in this case, the competition follows a non-monotonous scenario where the rate of change of cell numbers reverses during the simulation. After the barrier is removed, first motile cells invade the domain of non-motile cells until a certain stage where they are already in substantial majority. However, later the number of motile cells starts to decrease until they are completely eliminated. Thus, in this case, motility appears to provide an initial advantage until the cells are spatially separated, but as the invasion front and the distribution of cell types becomes more irregular the higher birth rate and lower death rate of the non-motile cells determine the outcome of competition and non-moving cells invade the whole domain.

**Fig. 9:**
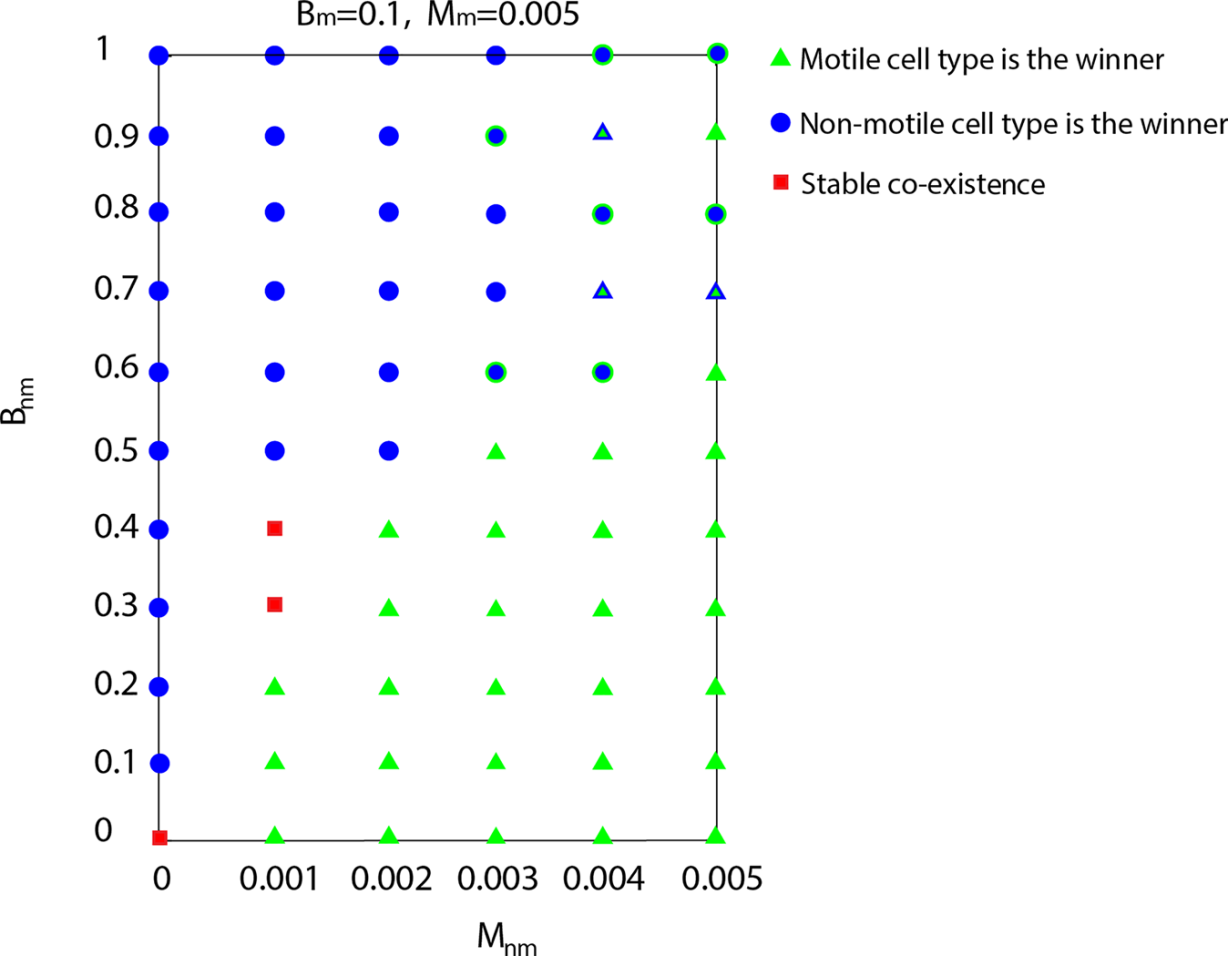
Phase diagram of the invasion process when the birth and death rates of the non-motile cells are varied. The parameters of the motile cells are fixed at $B_m=0.1$ and $M_m=0.005$. Each of the simulations was repeated three times. The symbols represent different simulation outcomes. Blue dots: non-motile cells win in all three simulations; green triangles: motile cells win every time; blue dots with green outline: non-motile cells win in two cases; green triangles with blue outline: non-motile cells win in two simulation runs; red squares: both cell types coexist in all simulations. The mechanical parameters are $\lambda _{cont}=0.5,\lambda _{area}=70,$ and $\lambda _{adh}=-10$

To capture the different types of outcomes, we ran a series of cell competition simulations where we varied both the birth rate and death rate of non-motile cells, whereas the parameters of motile cells were fixed at a relatively low birth rate $B_{m}=0.1$ and high death rate $M_m=0.005$ to balance out the inherent competitive advantage due to motility. To take into account the stochasticity of the Potts model, we repeated each simulation three times. The results are shown as a phase diagram in Fig. 9. Green symbols correspond to motile cells eliminating non-motile cells, and blue symbols represent the opposite case when non-motile cells win, in all three simulations. The blue dots with green outline indicate that in two out of three simulations the non-motile cells were the winners and the green triangles with blue outline represent the opposite case where the motile cells were the winners in two out of three simulations. As expected from the previous results, when there is a strong advantage in terms of higher birth rate and lower death rate of non-motile cells (top left corner) they can outcompete the motile cells; otherwise, motile cells win. Note, that when the death rates are the same ($M_m = M_{nm} = 0.005$) an increased birth rate alone is not sufficient to compensate for the advantage due to motility, and reduced death rate of the static cells is also necessary to change the outcome.

**Fig. 10:**
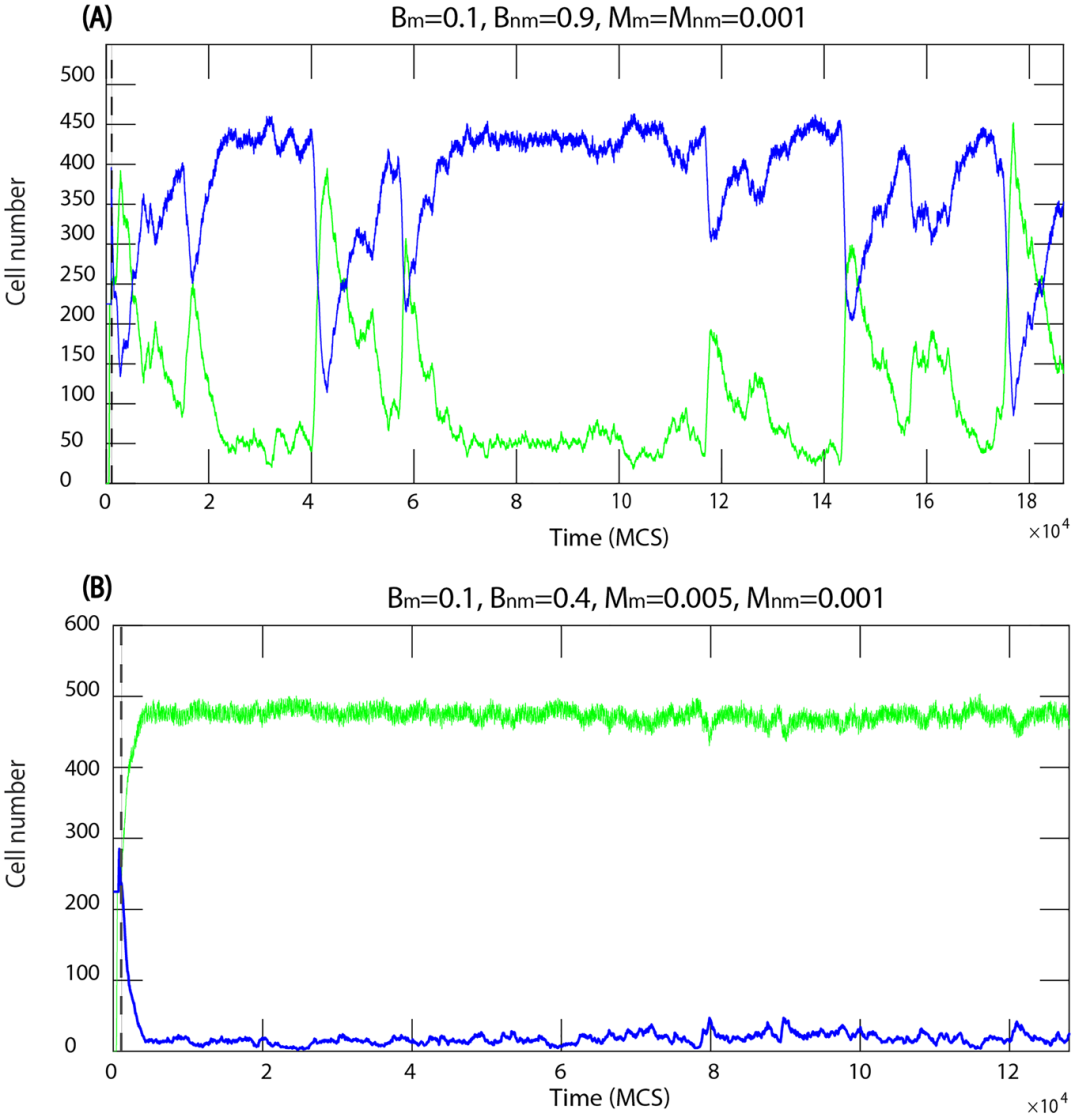
Number of motile and non-motile cells during the invasion, where the mechanical parameters are the same for both cell types. **A** and **B** demonstrate two qualitatively different examples of stable co-existence. The parameters in the Potts model energy function are $\lambda _{cont}=0.5,\lambda _{area}=70,$ and $\lambda _{adh}=-10$

Near the boundary between the two opposite outcomes, we also found cases of persistent coexistence of the two cell types (red squares in Fig. 9). Two typical examples for coexistence are shown in Fig. 10(A) and (B). In (B) the cell types coexist while each occupies an approximately constant proportion of the domain, while in the other case (A) the coexistence is much more dynamic following a sequence of alternating incomplete invasions by each cell type. In both cases, the coexistence appears to persist over a long time and neither type is eliminated.

## Discussion

Cells in a multicellular organism can gain a competitive advantage against others by genetic mutations and expand the space they occupy within a tissue. Cell motility can often contribute to the spread of such modified cells. In this paper, we investigated the role of cell motility in the mixing and competition of heterogeneous epithelial cell populations using a computational model.

The simulations demonstrate that active motility can enhance the mixing of different cell types and also confers an advantage in competitive cell invasion of otherwise identical cells. However, the effects of cell motility are dependent on the mechanical and biological properties of the cells. Mixing of motile cells into a population of non-moving cells can be inhibited by increased cell contractility creating a rigid barrier to mixing. Also, the advantage of motile cells in competitive invasion can be suppressed by differential proliferation and death rates. This may result in either the elimination of the motile cells or the stable coexistence of multiple cell types.

These above observations resulting from numerical simulations suggest a few potentially interesting predictions that could be investigated in cell culture experiments. Contractility of the cell cortex and/or proliferation rates could be manipulated by biochemical perturbations. Observing how these can influence the movement of the interface between different types of cells in such experiments may provide insights into potential treatment strategies for growing tumours.

Our simulations were restricted to two distinct spatially separated cell types. However, cancer cells often develop high genetic variability within a growing tumour. Therefore, it could be also interesting to investigate cell competition in a system with multiple mutations or with more general variability continuously generated through a stochastic process affecting the cell parameters. Such a model may provide insights into the process of cancer initiation.

## Supplementary Information

Below is the link to the electronic supplementary material.

Supplementary file 1: Simulation of mixing of motile (light blue or green) and non-motile (blue) cells with constant cell numbers (i.e. no cell division or death). Simulation parameters are: $\lambda _{\text {area}} = 70$, $\lambda _{\text {cont}} = 0.5$, and $\lambda _{\text {adh}} = -10$, and $F_{m}=1000$ (mp4 138980 KB)

Supplementary file 2: Simulation of partial mixing of motile and non-motile cells in a system with constant cell numbers. Simulation parameters are: $\lambda _{\text {area}} = 70$, $\lambda _{\text {cont}}^{nm} = 7$, $\lambda _{\text {cont}}^{m} = 0.5$, $\lambda _{\text {adh}} = -10$, and $F_{m}=400$ (mp4 223083 KB)

Supplementary file 3: Competitive invasion of motile cells eliminating non-motile cells. Simulation parameters are: $\lambda _{\text {area}} = 70$, $\lambda _{\text {cont}} = 0.5$, $M = 0.001$, $\lambda _{\text {adh}} = -10$, $F_{m}=1000$, and $B_{nm} =B_{m}= 0.9$ (mp4 301709 KB)

Supplementary file 4: Competitive invasion of motile cells initially invading most of the domain, but later reversed by non-motile cells with higher birth rate. Simulation parameters are: $\lambda _{\text {area}} = 70$, $\lambda _{\text {cont}} = 0.5$, $M = 0.003$, $\lambda _{\text {adh}} = -10$, $F_{m}=1000$, $B_{m} = 0.1$, and $B_{nm} = 0.9$ (mp4 328193 KB)

## Data Availability

No datasets were generated or analysed during the current study.
